# Supplementary material for: Genetic Evidence Prioritizes Neurocognitive Decline as a Causal Driver of Sleep Disturbances: A Multi-Omics Analysis Identifying Causal Genes and Therapeutic Targets
Source: Curr Issues Mol Biol. 2025 Nov 20;47(11):967. doi: 10.3390/cimb47110967 (PMC12651937; doi:10.3390/cimb47110967)
Supplement: Supplementary file 1 [file cimb-47-00967-s001.zip › cimb-3966126-supplementary/Supplementary Figures S1-S5-xQTLMR.pdf]

Supplementary Figures

Supplementary Figure S1. Comprehensive Results of the Bidirectional Mendelian Randomization Analysis. 2
Supplementary Figure S2. Leave-One-Out Sensitivity Analysis for Top Bidirectional Mendelian Randomization Associations. 4
Supplementary Figure S3. Funnel Plots for Assessing Directional Pleiotropy in Top Bidirectional MR Associations. 6
Supplementary Figure S4. Differential Gene Expression Profiles in Neurodegenerative and Sleep-Related Contexts. 8
Supplementary Figure S5. Comprehensive Single-Cell Expression Profiles of Key Candidate Genes in Alzheimer's Disease. 10

.

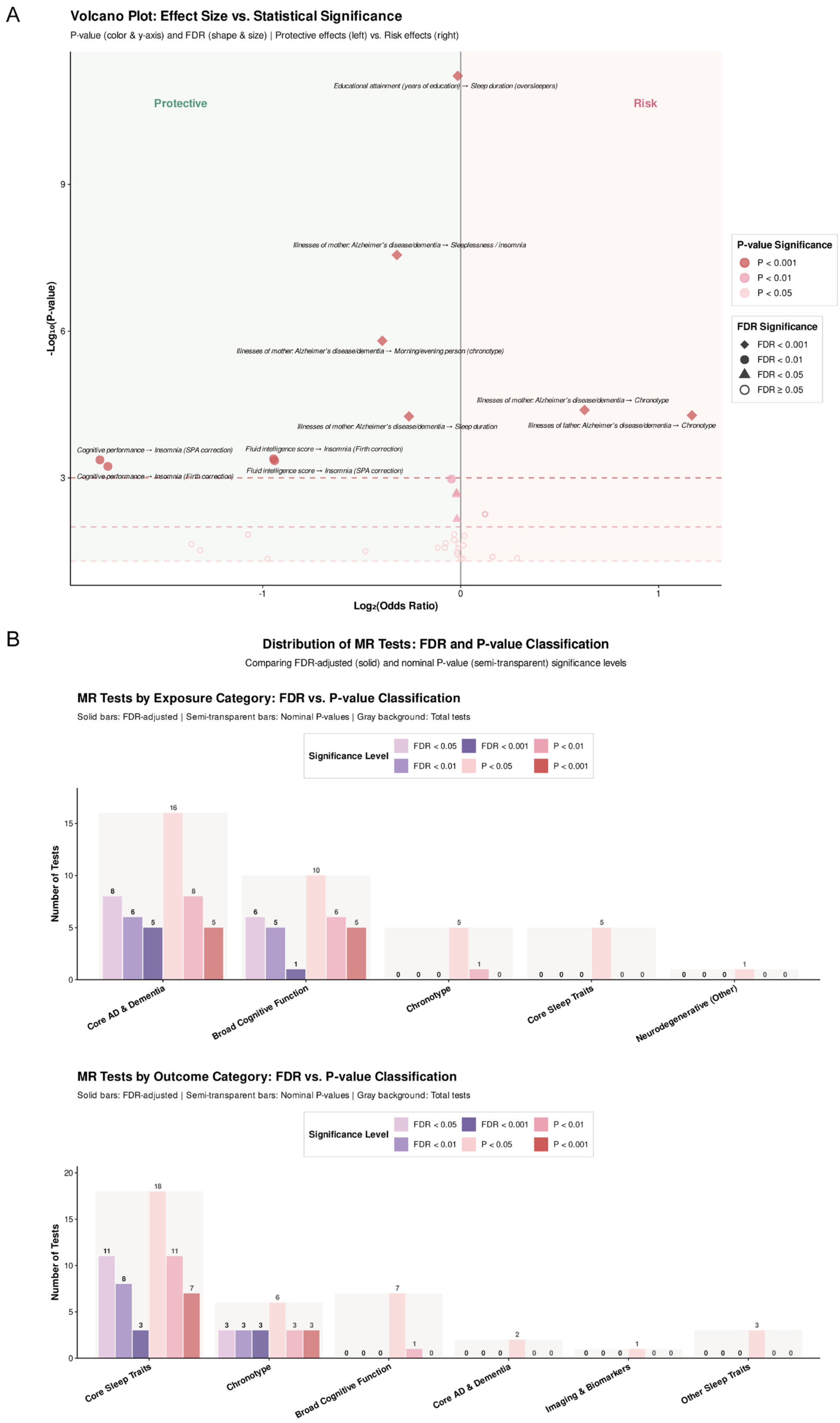

Supplementary Figure S1. Comprehensive Results of the Bidirectional Mendelian Randomization Analysis.

(A) Volcano plot of effect size versus statistical significance. This plot displays the $\log_2$(Odds Ratio) and $-\log_{10}$(*P*-value) for all tested causal associations, separated by

protective (left) and risk (right) effects. The color of each point indicates its nominal *P*-value significance, while the shape and size denote its false discovery rate (FDR) significance level. The plot clearly illustrates a strong enrichment of FDR-significant, protective associations for the causal pathway from neurocognitive traits to sleep traits. The top hit is the protective effect of higher educational attainment on the risk of being an over-sleeper (OR = 0.990, q = 3.84×10^{-10}). Other high-confidence findings include the protective effect of maternal AD/dementia liability on sleeplessness/insomnia (OR = 0.800, q = 8.75×10^{-7}).

(B) Distribution of MR tests by FDR and P-value classification. This panel quantifies the asymmetry in causal evidence. The top bar chart shows the number of significant MR tests when neurocognitive traits are used as the exposure, grouped by phenotypic category. The **'Core AD & Dementia'** category yielded the highest number of associations, with 8 passing FDR < 0.05. The bottom bar chart shows the results for the reverse direction, with sleep traits as the exposure. Here, the **'Core Sleep Traits'** category yielded the highest number of nominally significant associations (18 total), but only 3 of these survived FDR < 0.05 correction. This stark contrast between the number of FDR-significant hits in the forward direction (e.g., 8 from 'Core AD & Dementia') versus the reverse direction (0 from 'Chronotype' and 'Core Sleep Traits') provides a quantitative summary of the predominantly unidirectional causal flow from neurocognitive traits to sleep patterns.

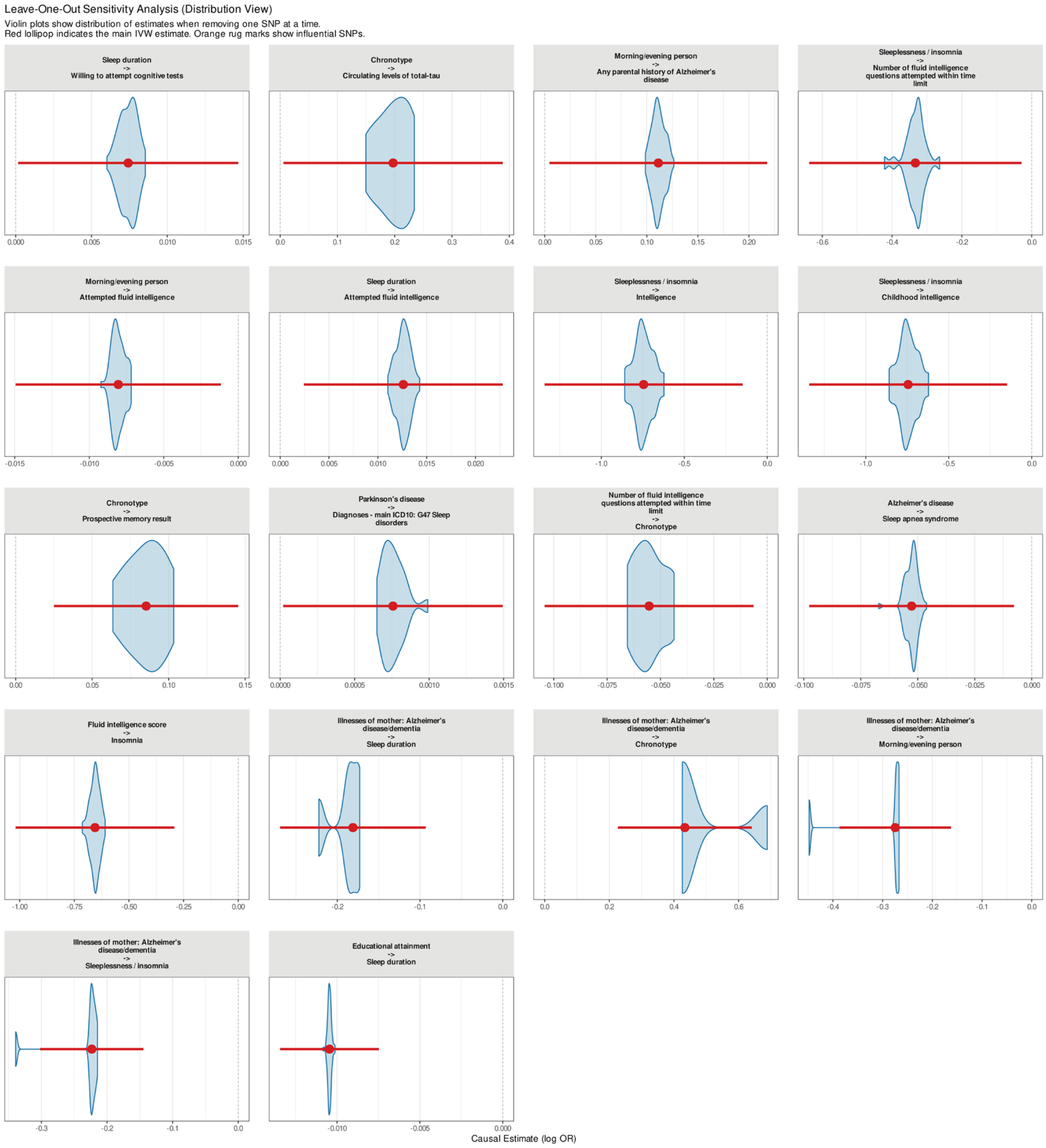

Supplementary Figure S2. Leave-One-Out Sensitivity Analysis for Top Bidirectional Mendelian Randomization Associations.

This figure presents the results of the leave-one-out sensitivity analysis for a selection of the most significant causal associations identified in the bidirectional MR analysis. Each panel corresponds to a specific exposure-outcome pair. The violin plot (blue) in each panel illustrates the distribution of the Inverse Variance Weighted (IVW) causal estimates when each instrumental SNP is systematically removed one at a time. The red point and line indicate the main IVW estimate calculated using all SNPs for that association. This analysis assesses the influence of individual SNPs on the overall causal estimate. Robust associations are indicated by a tight distribution of leave-one-out estimates that consistently remain on the same side of the null line (zero).

For example, the strong protective effect of maternal AD/dementia liability on sleeplessness/insomnia is shown to be highly robust, as the distribution of leave-one-out estimates is tightly clustered around the main IVW estimate and remains

consistently below zero. Similarly, the association between educational attainment and sleep duration (oversleepers) shows a very narrow distribution, indicating that the causal estimate is not driven by any single influential SNP. In contrast, some associations, such as the link between sleeplessness/insomnia and the number of fluid intelligence questions attempted, show a wider distribution, suggesting greater variability in the estimate depending on the specific SNP removed, although the overall direction of the effect remains consistent. Overall, the leave-one-out analysis supports the robustness of the primary causal associations presented in the main text, confirming that they are not unduly influenced by single, highly pleiotropic genetic variants.

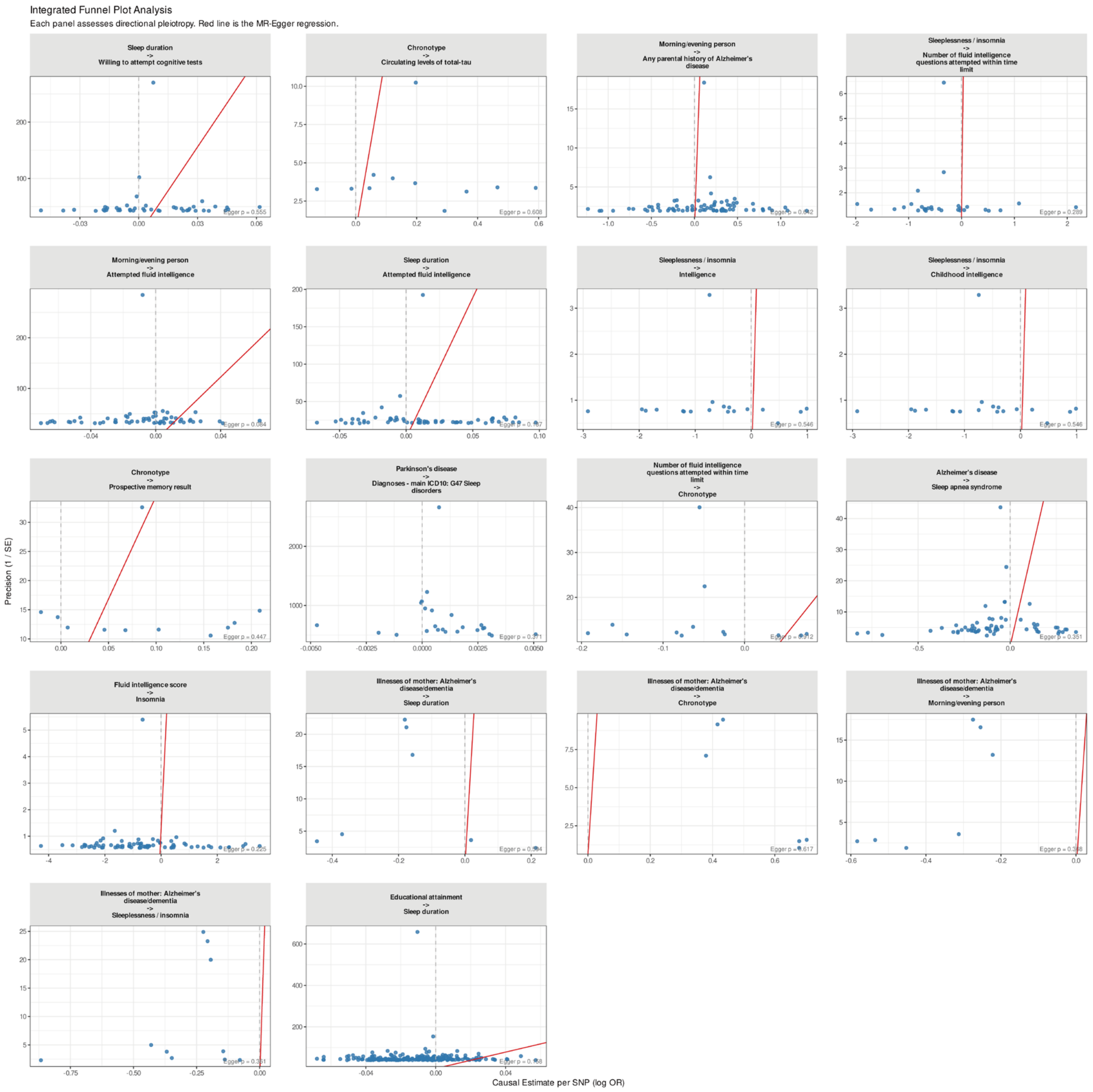

Supplementary Figure S3. Funnel Plots for Assessing Directional Pleiotropy in Top Bidirectional MR Associations.

This figure presents funnel plots for a selection of the most significant causal associations identified in the bidirectional MR analysis. Each panel corresponds to a specific exposure-outcome pair. The x-axis represents the causal estimate for each individual SNP (log OR), and the y-axis represents the precision of the estimate (1 / Standard Error). In the absence of directional pleiotropy, the SNPs are expected to be symmetrically distributed around the main causal estimate. The red line represents the regression line from the MR-Egger analysis, and its intercept with the y-axis provides a formal test for directional pleiotropy. A significant deviation of the intercept from zero (indicated by the MR-Egger p-value) suggests the presence of pleiotropy.

The majority of the high-confidence associations in the neurocognitive-to-sleep direction, such as the link between "Educational attainment" and "Sleep duration," display a symmetrical distribution of SNPs around the central estimate, with a non-significant MR-Egger intercept (p = 0.584), supporting the validity of the causal estimate. Similarly, the association between "Fluid intelligence score" and "Insomnia"

also shows a symmetrical pattern (*p* = 0.447). An exception is the association between "Illnesses of mother: Alzheimer's disease/dementia" and "Sleeplessness / insomnia," which, despite a highly significant primary IVW estimate, shows a significant MR-Egger intercept (*p* = 0.001), indicating that the causal estimate should be interpreted with caution due to potential pleiotropic effects. Overall, these funnel plots provide visual and statistical support for the robustness of most of our primary findings against directional pleiotropy.

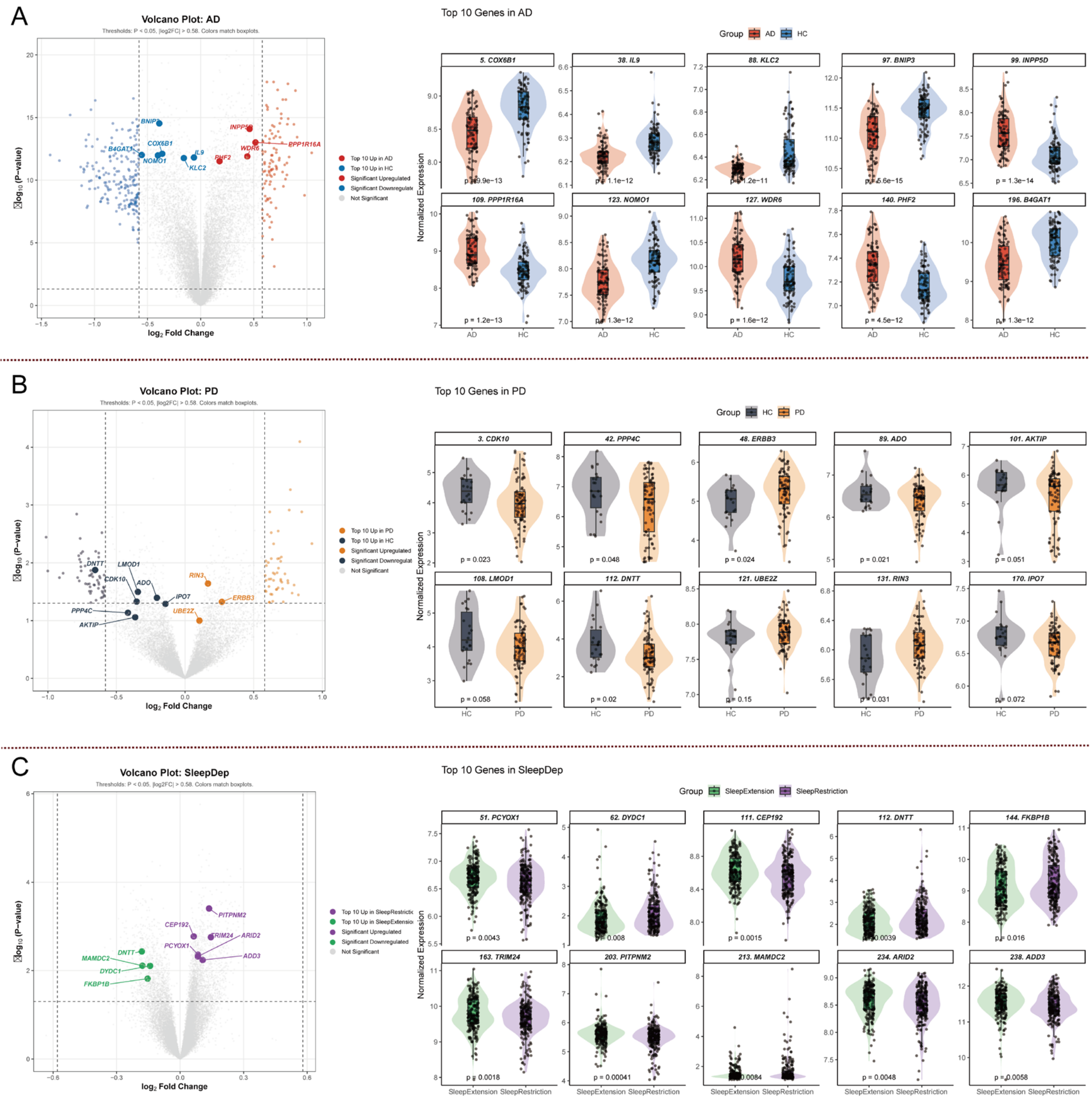

Supplementary Figure S4. Differential Gene Expression Profiles in Neurodegenerative and Sleep-Related Contexts.

This figure presents the results of differential gene expression analysis for three independent conditions, with each panel containing a volcano plot for a global overview and violin plots detailing the expression of the top 10 differentially expressed genes.

(A) Differential expression in Alzheimer's Disease (AD) versus healthy controls (HC). The volcano plot on the left visualizes global expression changes, highlighting significantly upregulated (red) and downregulated (blue) genes. The violin plots on the right show the expression distributions for the top 10 hits. A strong and widespread dysregulation is evident, with genes such as *COX6B1* ($p = 9.0 \times 10^{-13}$), *IL9* ($p = 1.1 \times 10^{-12}$), and *KLC2* ($p = 1.2 \times 10^{-11}$) being significantly downregulated in AD. Conversely, *BNIP3* ($p = 6.6 \times 10^{-15}$) and *INPP5D* ($p = 1.3 \times 10^{-14}$) were among the most significantly upregulated genes, indicating a robust transcriptomic signature in the AD brain.

(B) Differential expression in Parkinson's Disease (PD) versus healthy controls. The volcano plot identifies significantly dysregulated genes (upregulated in orange,

downregulated in blue), and the violin plots detail the expression of the top 10 hits. Compared to AD, the expression changes in PD were more modest but still significant for several genes. For example, *CDK10* (p = 0.023) and *PPP4C* (p = 0.048) were significantly downregulated, while *ERBB3* (p = 0.024) and *ADO* (p = 0.021) were significantly upregulated in PD brains.

(C) Differential expression between Sleep Restriction and Sleep Extension conditions. The volcano plot shows genes downregulated (green) or upregulated (purple) in the sleep restriction group, with the top 10 genes detailed in the violin plots. This analysis revealed a distinct signature of sleep deprivation, characterized by the significant downregulation of multiple genes, including *PCYOX1* (p = 0.0043), *DYDC1* (p = 0.008), *PITPNM2* (p = 0.00041), and *TRIM24* (p = 0.0018). These findings highlight a specific set of genes whose expression is sensitive to sleep state.

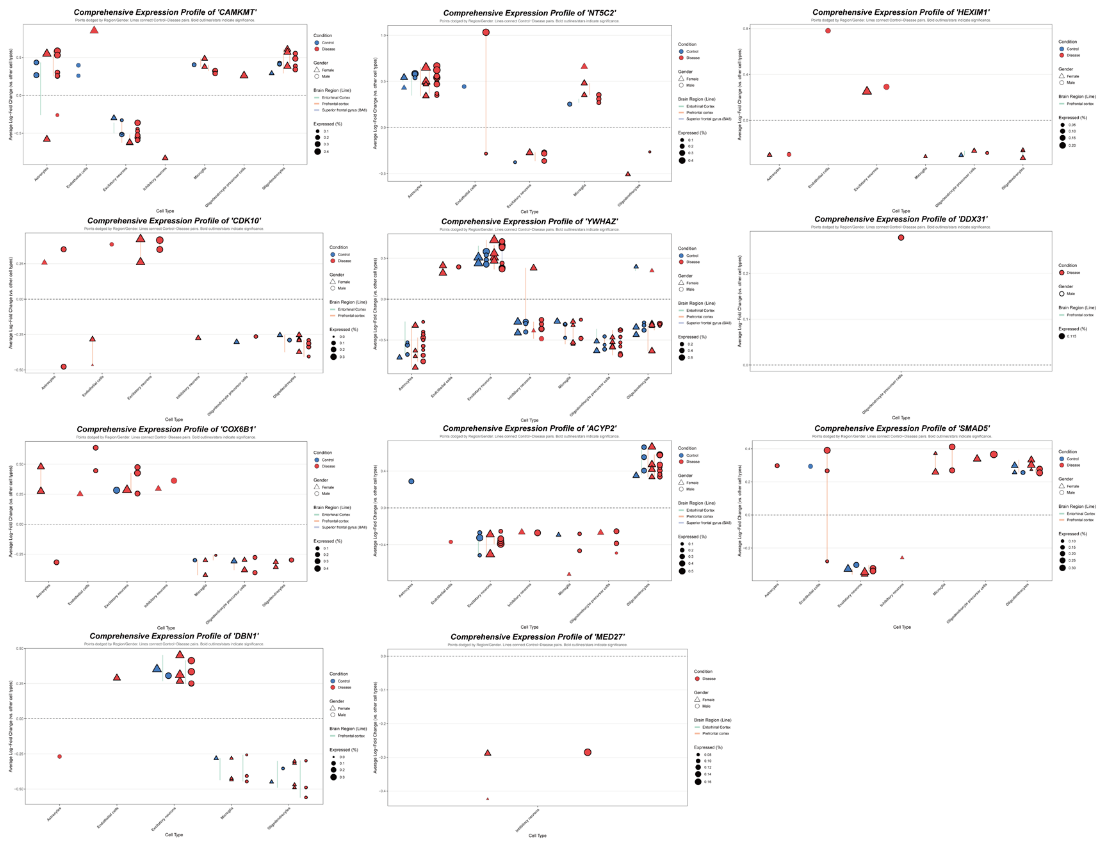

Supplementary Figure S5. Comprehensive Single-Cell Expression Profiles of Key Candidate Genes in Alzheimer's Disease.

This figure provides a detailed visualization of the expression profiles for 11 key candidate genes across multiple brain cell types, stratified by condition (Control vs. Disease), brain region, and sex. Each panel is dedicated to a single gene. The y-axis represents the average log-fold change (logFC) of the gene's expression in a specific cell type compared to the average expression across all other cell types. The x-axis categorizes the data by cell type. Points are colored by condition (blue for Control, red for Disease) and shaped by sex (circle for Female, triangle for Male). The size of each point is proportional to the percentage of cells within that group expressing the gene. Orange vertical lines connect points from the same sub-context (e.g., the same cell type, region, and sex) to highlight the shift in expression between the control and disease states.

The plots reveal complex and highly specific expression patterns. For example, the panel for *YWHAZ* shows its high baseline expression in Excitatory neurons (positive logFC) and low baseline expression in glial cells like Astrocytes and Microglia (negative logFC). In the disease state, this pattern is amplified, with expression increasing further in neurons and decreasing further in glia. The panel for *CAMKMT* illustrates a strong opposing regulation, with the gene being highly expressed in Oligodendrocytes (positive logFC) but lowly expressed in Excitatory neurons (negative logFC). In disease, this divergence is exacerbated, with expression increasing in oligodendrocytes and decreasing in neurons. Other genes, such as *COX6B1*, show a more restricted high-expression profile, being particularly enriched in Endothelial cells. This granular visualization underscores the cellular and contextual heterogeneity of gene expression in the Alzheimer's disease brain.
